# Machine learning and metabolomics identify biomarkers associated with the disease extent of ulcerative colitis

**DOI:** 10.1093/ecco-jcc/jjaf020

**Published:** 2025-02-04

**Authors:** Changchang Ge, Yi Lu, Zhaofeng Shen, Yizhou Lu, Xiaojuan Liu, Mengyuan Zhang, Yijing Liu, Hong Shen, Lei Zhu

**Affiliations:** Department of Gastroenterology, Jiangsu Provincial Hospital of Chinese Medicine, Affiliated Hospital of Nanjing University of Chinese Medicine, Nanjing 210029, China; Department of Gastroenterology, Jiangsu Provincial Hospital of Chinese Medicine, Affiliated Hospital of Nanjing University of Chinese Medicine, Nanjing 210029, China; Department of Gastroenterology, Jiangsu Provincial Hospital of Chinese Medicine, Affiliated Hospital of Nanjing University of Chinese Medicine, Nanjing 210029, China; Department of Gastroenterology, Jiangsu Provincial Hospital of Chinese Medicine, Affiliated Hospital of Nanjing University of Chinese Medicine, Nanjing 210029, China; Department of Gastroenterology, Jiangsu Provincial Hospital of Chinese Medicine, Affiliated Hospital of Nanjing University of Chinese Medicine, Nanjing 210029, China; Department of Gastroenterology, Jiangsu Provincial Hospital of Chinese Medicine, Affiliated Hospital of Nanjing University of Chinese Medicine, Nanjing 210029, China; Department of Gastroenterology, Jiangsu Provincial Hospital of Chinese Medicine, Affiliated Hospital of Nanjing University of Chinese Medicine, Nanjing 210029, China; Department of Gastroenterology, Jiangsu Provincial Hospital of Chinese Medicine, Affiliated Hospital of Nanjing University of Chinese Medicine, Nanjing 210029, China; Department of Gastroenterology, Jiangsu Provincial Hospital of Chinese Medicine, Affiliated Hospital of Nanjing University of Chinese Medicine, Nanjing 210029, China

**Keywords:** ulcerative colitis, metabolomics, machine learning

## Abstract

**Background and aims:**

Ulcerative colitis (UC) is a metabolism-related chronic intestinal inflammatory disease. Disease extent is a key parameter of UC. Using serum metabolic profiling to identify noninvasive biomarkers of disease extent may inform therapeutic decisions and risk stratification.

**Methods:**

The orthogonal partial least squares–discriminant analysis (OPLS-DA) was performed to identify the metabolites. Least absolute shrinkage and selection operator regression, random forest-recursive feature elimination, and support vector machine-recursive feature elimination algorithms were used to screen metabolites. Five machine learning algorithms (eXtreme Gradient Boosting, K-NearestNeighbor, Naive Bayes, random forest [RF], and SVM) were used to construct the prediction model.

**Results:**

A total of 220 differential metabolites between the patients with UC and healthy controls (HCs) were confirmed by the OPLS-DA model. Machine learning screened 8 essential metabolites for distinguishing patients with UC from HCs. A total of 23, 6, and 6 differential metabolites were obtained through machine learning between groups E1 and E2, E1 and E3, and E2 and E3. The RF model had a prediction accuracy of up to 100% in all 3 training sets. The serum levels of tridecanoic acid were significantly lower, and pelargonic acid was significantly higher in patients with extensive colitis than in the other groups. The serum level of asparaginyl valine in patients with rectal UC was significantly lower than that in the E2 and E3 groups.

**Conclusions:**

Our findings revealed the metabolic landscape of UC and identified biomarkers for different disease extents, confirming the value of metabolites in predicting the occurrence and progression of UC.

## Graphical Abstract

**Graphical Abstract:**
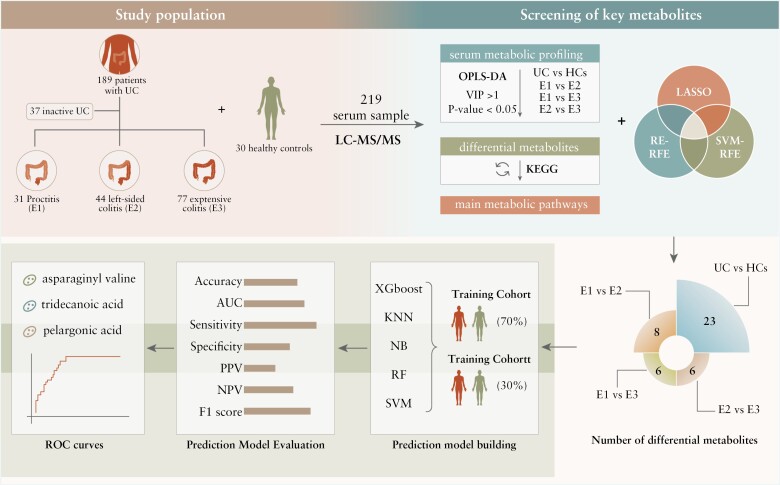

## 1. Introduction

Ulcerative colitis (UC) is a chronic nonspecific intestinal inflammatory disease and a subtype of inflammatory bowel disease (IBD) characterized by recurrent diarrhea, bloody stools, and abdominal pain. In the 21st century, globally, the incidence of UC is increasing rapidly.^1^ The cause of UC is not clear, but it is generally believed that environmental factors and intestinal microbial components can stimulate the immune response of genetically susceptible individuals, interfering with energy metabolism in immune cells of the gastrointestinal tract and disrupting intestinal microbial metabolism.^2,3^ We can observe heterogeneity at the clinical, genetic, and molecular levels, which is mainly manifested in individual metabolome differences.

Metabolomics, a high-throughput tool for quantitatively analyzing small-molecule metabolites in biospecimens such as blood, tissue, urine, and saliva, enables us to study the metabolic alterations caused by UC.^4^ Di’Narzo et al. analyzed the serum metabolites of patients with UC and Crohn’s disease (CD) and found that several metabolites associated with disease activity can be used to distinguish patients with IBD from healthy people.^5^ Hua et al. found that patients with IBD had metabolic pathway disorders before diagnosis. Changes in unsaturated fatty acid biosynthesis and fatty acid biosynthesis–related metabolic pathways are associated with UC, and most of the associated metabolites are negatively related to the risk of UC.^6^ Metabolomics provides information on metabolite concentration changes, and it has great potential as a tool for discovering possible biomarkers and related metabolic pathways, as well as for exploring the complex pathophysiological mechanisms of diseases.

According to a gene association analysis study in The Lancet, there are significant differences in the genetics among UC, colonic CD, and ileal CD. Disease extent is an intrinsic aspect of disease that is in part genetically determined and is the major driver of changes in disease behavior over time.^7^ Atreya et al. summarized the clinical behavior, epidemiology, genetics, and intestinal microbial groups and demonstrated that the disease behavior of colonic CD overlapped with that of UC.^8^ In addition, numerous studies have shown that patients with CD with different lesion sites have different responses to biological agents. Meta-analysis results showed that in a randomized controlled trial, all biological agents tested had a better effect on colonic CD than on UC.^9,10^

Compared to CD lesion sites, the lesion sites of UC have received less research attention. UC lesion sites are considered to be confined to the colon, and the boundaries between different parts are not evident.^11^ Nevertheless, the Montreal classification divides UC into ulcerative proctitis (E1), left-sided colitis (E2), and extensive colitis (E3) according to the maximal extent upon follow up.^12^ This classification system is useful in ascribing phenotypes to patients, both for treatment purposes and to assist with service delivery and research. A retrospective study found differences in glucose, lipid, and protein metabolism-related indicators between distal colitis and extensive colitis at the same level of disease activity.^13^ However, the metabolomic profiles of patients with UC with different disease extents are unknown.

Although metabolomics can measure hundreds of metabolites in clinical samples, the complex data processing and interpretation remain a challenge. In recent years, advanced machine learning algorithms have been widely used to screen medical biomarkers and effectively identify disease-related metabolite markers. Therefore, for the first time, this study combines non-target metabolomics technology with machine learning algorithms to clarify the metabolomic characteristics of UC with different disease extents and identify potential biomarkers, providing a basis for the precise treatment of UC and informing patient risk stratification.

## 2. Materials and methods

### 2.1. Study populations

Patients with UC were recruited by the gastroenterology department of Jiangsu Provincial Hospital of Chinese Medicine from January 2019 to April 2022, and healthy controls (HCs) were enrolled through advertisements. This study has passed the ethics review of Jiangsu Provincial Hospital of Traditional Chinese Medicine (ethics review number: 2022NL-062-01), and written informed consent has been obtained from all eligible participants or their legal representatives.

All patients with UC were diagnosed according to a combination of UC clinical, endoscopic, and histopathological criteria. Age, sex, height, weight, course of disease, location and extent of disease, smoking status, and colonoscopy reports were recorded for each patient (Table S1). The Montreal classification was used to assess the extent. The Montreal classification of disease extent of UC allows the extent to be defined into 3 subgroups, and there are: ulcerative proctitis (involvement limited to the rectum, E1), left-sided UC (involvement limited to a proportion of the colorectum distal to the splenic flexure, E2), and extensive UC (involvement extends proximal to the splenic flexure, E3).^12^ Based on the above criteria, we grouped patients according to the maximal macroscopic inflammation that could be observed under colonoscopy. The Mayo score was used to assess disease activity.^14^ The inactive disease inclusion criteria were rectal bleeding remission, normal defecation frequency, and total Mayo score < 3 or partial Mayo score < 2. Those who did not meet the above criteria were judged as having active UC. The exclusion criteria included age under 18 years, pregnancy, history of colorectal surgery, active infections, and current malignancy. The HCs were selected and matched to the patients based on age and sex. They had no current or history of severe physical disease.

### 2.2. Chemicals, reagents, and equipment

Methanol, acetonitrile, ammonium acetate (NH4Ac), and ethanoic acid (AcOH) were all liquid chromatograph–mass spectrometer (LC-MS) grade and were purchased from CNW Technologies, SIGMA-ALDRICH, and Fisher Chemical, respectively. All the experiments were conducted on an ultra-high performance liquid chromatography (UHPLC) system (Vanquish, Thermo Fisher Scientific) with a UPLC BEH Amide column (1.7 μm, 2.1 mm × 100 mm) coupled to an Orbitrap Exploris 120 mass spectrometer (Orbitrap MS, Thermo). The ACQUITY UPLC BEH Amide column (1.7 μm, 2.1 mm × 100 mm) was purchased from Waters Corporation.

### 2.3. Serum sample collection and metabolite extraction

#### 2.3.1. Serum sample collection

For each patient, serum samples were collected for metabolomics analysis. The serum was separated from the peripheral blood samples in eppendorf tubes by centrifuging at 1000 *g* for 10 minutes at 4 °C (centrifugal radius = 10 cm) and immediately stored at −80 °C until future metabolomics analysis to minimize the metabolic degradation process.

#### 2.3.2. Metabolite extraction

First, 50 μL of a sample was transferred to an EP tube. After the addition of 200 μL of extract solution (acetonitrile:methanol = 1:1, containing an isotopically labeled internal standard mixture), the samples were vortexed for 30 seconds, sonicated for 10 minutes in an ice water bath, and incubated for 1 hour at −40 °C to precipitate proteins. Then, the sample was centrifuged at 12 000 rpm (relative centrifugal force [RCF] = 13 800 *g*, centrifugal radius = 8.6 cm) for 15 minutes at 4 °C.^15^ The resulting supernatant was transferred to a fresh glass vial for analysis. The quality control sample was prepared by mixing equal aliquots of the supernatants from all of the samples.

### 2.4. Liquid chromatography-tandem mass spectrometry analysis and data preprocessing

#### 2.4.1. Liquid chromatography-tandem mass spectrometry analysis

Liquid chromatography-tandem mass spectrometry analyses were performed using a UHPLC system (Vanquish, Thermo Fisher Scientific) with a UPLC BEH Amide column (1.7 μm, 2.1 mm × 100 mm) coupled to Orbitrap Exploris 120 mass spectrometer (Orbitrap MS, Thermo). The mobile phase consisted of 25 mmol/L ammonium acetate and 25 mmol/L ammonium acetate in water (pH = 9.75) (A) and acetonitrile (B). The auto-sampler temperature was 4 °C, and the injection volume was 2 μL.

The Orbitrap Exploris 120 mass spectrometer was used for its ability to acquire MS/MS spectra in information-dependent acquisition mode, controlled by the acquisition software (Xcalibur, Thermo). In this mode, the acquisition software continuously evaluates the full scan MS spectrum. The ESI source conditions were set as follows: sheath gas flow rate, 50 Arb; Aux gas flow rate, 15 Arb; capillary temperature, 320 °C; full MS resolution, 60 000; MS/MS resolution, 15 000; collision energy, 10/30/60 in normalized collision energy (NCE) mode; and spray voltage, 3.8 kV (positive) or −3.4 kV (negative).

#### 2.4.2. Data preprocessing

X peaks were detected, and X metabolites were left after relative standard deviation de-noising. Then, we preprocess the original data, which mainly includes the following 4 steps: (1) Deviation value filtering: deviation value filtering is performed on a single Peak based on the relative standard deviation, that is, the coefficient of variation; (2) missing value filtering: only retain the peak area data with no more than 50% null value in a single group or no more than 50% null value in all groups; (3) missing value filling: simulate the missing values in the original data and fill them with half of the minimum value; (4) data normalization processing: the total ion current of each sample is used for normalization. In the end, after preprocessing, 10 426 peaks were retained. Then, an in-house MS2 database (BioTree DB) was applied for metabolite annotation. The cutoff for annotation was set at 0.3.

### 2.5. Statistical analysis

The final dataset, containing the peak number, sample name, and normalized peak area, was imported into the SIMCA16.0.2 software package (Sartorius Stedim Data Analytics AB) for multivariate analysis. Orthogonal projections to latent structures-discriminant analysis (OPLS-DA) was applied to visualize group separation and screen for effective differential metabolites. OPLS-DA is a powerful multivariate analysis tool, which is often used to extract taxonomic information from high-dimensional data, especially in metabolomics, genomics, and other fields. The characteristic of this method is that it can remove the metabolites independent of grouping factors, and makes the grouping information mainly concentrated in one principal component, this makes the model simple and easy to interpret.^16^In order to verify the robustness of the OPLS-DA model and avoid overfitting, we used a 7-fold cross-validation and performed 200 permutations, then the validity of the model is evaluated by R^2^Y (the interpretability of the model to the categorical variable Y) and Q^2^ (the predictability of the model). The closer the 2 values are to one, the more reliable the model is. In the OPLS-DA model, the variable influence on projection (VIP) values presents the overall effect of the variable on the grouped variables. Metabolites with VIP > 1 and *P* < .05 (Student’s *t*-test) were considered significantly changed metabolites. Information on the identified differential metabolites was then fed into MetaboAnalyst (https://www.metaboanalyst.ca/MetaboAnalyst/) to perform Kyoto Encyclopedia of Genes and Genomes (KEGG) enrichment analysis and obtain significantly perturbed metabolic pathways.

Next, we used 3 machine learning methods to screen metabolites, namely, the least absolute shrinkage and selection operator (LASSO), random forest-recursive feature elimination (RF-RFE), and support vector machine-recursive feature elimination (SVM-RFE) algorithms. The LASSO algorithm constructs a penalty function to obtain a more refined model; when fewer predictors are desired, LASSO regression is the preferred option to reduce model dimensionality.^17^ Compared with classical statistical methods, LASSO regression has shown better performance for prediction model selection and better identification of predictors.^18–20^ The RF-RFE and SVM-RFE algorithms were also used to screen differential metabolites. To improve the reliability of the results, all distinctive features were fed into the LASSO, RF-RFE, and SVM-RFE algorithms in the form of 10-fold cross-validation. The differential metabolites screened by the 3 machine learning algorithms were then intersected to further narrow the range of metabolites and build predictive models.

**Figure 1.**
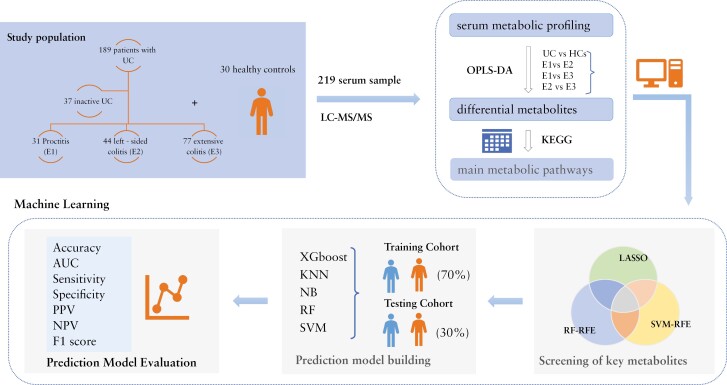
The overall process of the study.AUC, area under the receiver operating characteristic curve; KEGG, Kyoto Encyclopedia of Genes and Genomes; KNN, K-NearestNeighbor; LASSO, least absolute shrinkage and selection operator; LC-MS/MS, liquid chromatography-tandem mass spectrometry; NB, Naive Bayes; NPV, negative predictive value; OPLS-DA, orthogonal projections to latent structures-discriminant analysis; PPV, positive predictive value; RF, random forest; RFE, recursive feature elimination; SVM, support vector machine; UC, ulcerative colitis; Xgboost, eXtreme Gradient Boosting.

Based on the potential key metabolites, we used 5 machine learning algorithms (eXtreme Gradient Boosting [XGboost], K-NearestNeighbor [KNN], Naive Bayes [NB], RF, and SVM) to construct prediction models. Machine learning training was performed on 70% of the subset of the total column and verified on 30% of the reserved subset. This study evaluated the model using 7 evaluation metrics, namely, accuracy, sensitivity, specificity, positive predictive value, negative predictive value, F1 score, and area under the receiver operating characteristic curve (AUC). All machine learning methods were performed using R software (version 4.3.2). Figure 1 shows the overall process of the study.

## 3. Results

### 3.1. Clinical characteristics of the study population

A total of 189 patients with UC (152 active UC and 37 inactive UC) and 30 HCs were included in this study, and the patients with active UC were divided into E1, E2, and E3 groups. The HCs had a median age of 38.6 years and comprised 17 males and 13 females. The clinical characteristics of patients with UC are listed in Table 1. There were no differences in the baseline data of patients in each group in terms of sex, age, disease severity, treatment, or body mass index.

**Table 1.** Clinical characteristics of patients with ulcerative colitis.

	E1 (*N* = 31)	E2 (*N* = 44)	E3 (*N* = 77)	Inactive UC (*N* = 37)	*P-*value ^a^
Male, *n* (%)	12 (38.7%)	26 (59.1%)	44 (57.1%)	21 (56.8%)	.286
Age, y (median and range)	42 (32–55)	41.5 (34.25–52)	44 (29–49.5)	44 (36-51)	.781
Disease activity, *n* (%)					
Remission				37	
Mild	23 (74.2%)	20 (45.5%)	44 (57.1%)		.113
Moderate	8 (25.8%)	21 (47.7%)	27 (35.1%)		
Severe	0	3 (6.8%)	6 (7.8%)		
Treatment, *n* (%)					
5-Aminosalicylic acid	29 (93.5%)	43 (97.7%)	76 (98.7%)	33 (89.2%)	.095
Corticosteroid	1 (3.2%)	3 (6.8%)	4 (5.2%)	0	.455
Immunosuppressant	0 (0.0%)	1 (2.3%)	2 (2.6%)	0	.630
Biological agents	1 (3.2%)	2 (4.5%)	2 (2.6%)	0	.645
BMI, kg/m^2^ (mean ± SD)	22.48 (3.01)	22.24 (2.69)	21.33 (2.95)	22.07 (3.67)	.359
Smoker, *n* (%)	4 (12.9%)	7 (15.9%)	6 (7.8%)	9 (24.3%)	.112

^a^We used Kruskal-Wallis tests and analysis of variance for numerical data and the χ^2^ test for categorical data.

BMI, body mass index; E1, ulcerative proctitis; E2, left-sided colitis; E3, extensive colitis.

### 3.2. Identification of differential metabolites between patients with UC and HCs

#### 3.2.1. OPLS-DA model construction

**Figure 2.**
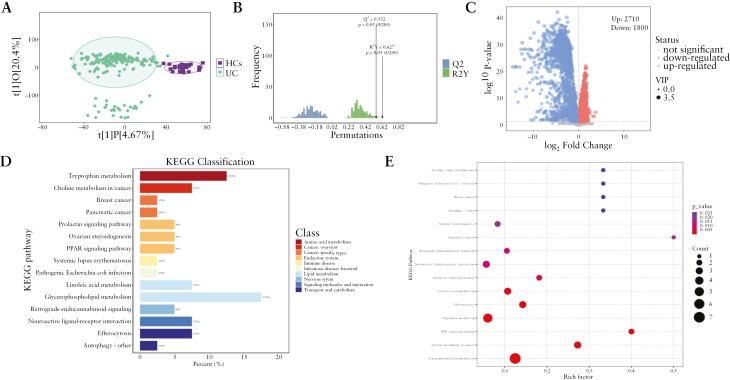
(A) OPLS-DA score scatter plot between the UC group and the HC group; (B) the permutation test with a permutation number of 200 for the OPLS-DA model; (C) volcano plot for the UC group and the HC group; (D) KEGG classification for differential metabolites between the UC group and the HC group; (E) KEGG enrichment for differential metabolites between the UC group and the HC group. HC, healthy control; KEGG, Kyoto Encyclopedia of Genes and Genomes; OPLS-DA, orthogonal projections to latent structures-discriminant analysis; UC, ulcerative colitis.

The OPLS-DA model was used to compare the metabolic profile differences between the UC group and the HC group. Figure 2A shows the visual and intuitive results of the supervised OPLS-DA analysis of metabolic profiles. The OPLS-DA model exhibited obvious discrimination between the UC and HC groups. The permutation plot test of the OPLS-DA model for the UC and HC groups was reliable and robust, avoiding overfitting (Figure 2B). A 200-times permutation was conducted to validate the classification performance of the OPLS-DA model. The fit metrics values were R^2^Y = 0.627 (*P* < .01) and Q^2^ = 0.552 (*P* < .01), indicating that the OPLS-DA model constructed by serum metabolites can serve as an objective tool for the diagnosis of UC.

#### 3.2.2. Differential metabolites and KEGG enrichment analysis

A total of 19 951 substances were detected in the positive and negative ion modes. Of the 19 951 substances, 913 metabolites can be obtained by qualitative matching analysis of secondary mass spectrometry and have corresponding names that can be used for follow-up studies. After screening with VIP > 1 and *P*-value < .05, 2710 substances were found to be upregulated and 1800 substances were downregulated, of which 220 substances (137 upregulated substances and 83 downregulated) were identified (Figure 2C). The specific VIP score and *P*-value of each aforementioned metabolite are presented in Table S2.

KEGG pathway enrichment analyses of these differential metabolites revealed a range of perturbed metabolic pathways, mainly involving tryptophan metabolism, glycerophospholipid metabolism, and the peroxisome proliferator-activated receptor (PPAR) signaling pathway (Figure 2D and E, Table S3).

#### 3.2.3. Constructing UC disease prediction models using machine learning

**Figure 3.**
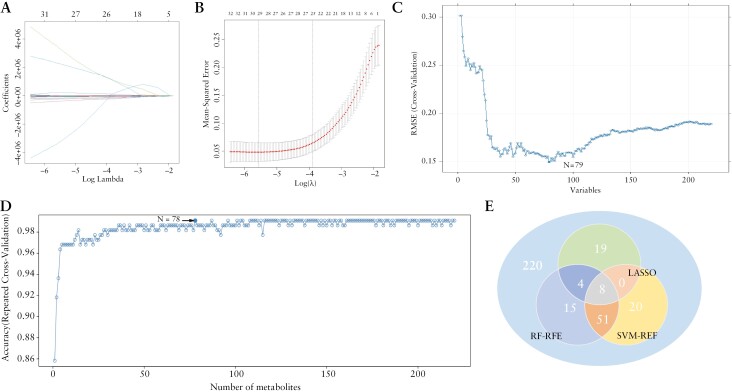
(A) LASSO coefficient path: The x-axis represents the value of the regularization parameter, and the y-axis represents the size of the model parameter. (B) LASSO regression cross-validation curve: The x-axis represents the value of the regularization parameter, and the y-axis represents the mean square error (MSE) of the model. The lowest point of the curve corresponds to the optimal regularization parameter value (lambda.min). (C) SVM-RFE algorithm variable screening diagram. (D) RF-RFE algorithm variable screening diagram. (E) The intersection differential metabolites of the LASSO regression algorithm, RF algorithm, and SVM-RFE algorithm. LASSO, least absolute shrinkage and selection operator; SVM-RFE, support vector machine-recursive feature elimination.

Based on the above 220 differential metabolites, the metabolic landscapes of HCs and patients with UC were compared, and the association between metabolic characteristics and disease was studied using machine learning algorithms. We used LASSO, RF-RFE, and SVM-RFE to screen the metabolites, and 8 essential metabolites (Figure 3) were selected to discriminate between patients with UC and HCs, namely, leucyl phenylalanine, 2-acetylthiazole, lithocholic acid glycine conjugate, eupatilin, N-acetyl-5-aminosalicylic acid, 5-hydroxy-7-(4-hydroxyphenyl)-1-phenyl-3-heptanone, octyl phenylacetate, and 3-beta-hydroxy-4-beta-methyl-5-alpha-cholest-7-ene-4-alpha-carbaldehyde.

**Figure 4.**
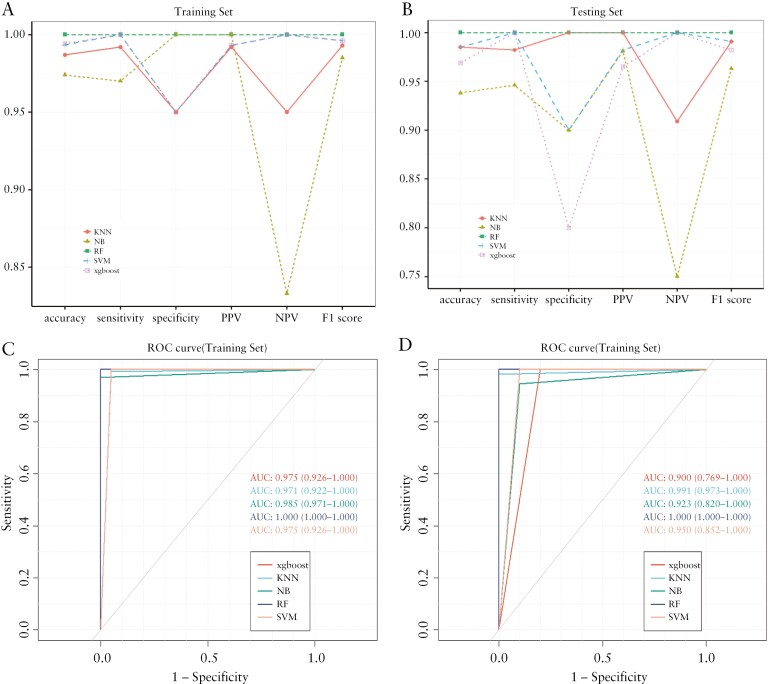
(A) The accuracy, sensitivity, specificity, PPV, NPV, and F1 score of the training set prediction model of UC. (B) The accuracy, sensitivity, specificity, PPV, NPV, and F1 score of the testing set prediction model of UC. (C) The ROC curve of the training set prediction model of UC. (D) The ROC curve of the testing set prediction model of UC. AUC, area under the receiver operating characteristic curve; KNN, K-NearestNeighbor; NB, Naive Bayes; NPV, negative predictive value; PPV, positive predictive value; RF, random forest; ROC, receiver operating characteristic; SVM, support vector machine; Xgboost, eXtreme Gradient Boosting.

To evaluate the predictive performance of these essential metabolites, we randomly divided 219 samples into a training set and a testing set in a 3:7 ratio and trained 5 machine learning models using the XGboost, KNN, NB, RF, and SVM algorithms (Figure 4). The model constructed by the RF algorithm exhibited very good performance in both the training and testing sets, and the sensitivity, specificity, and AUC were all 1. The other 4 models also had good predictive performances, confirming that these metabolites can be used as potential biomarkers for UC recognition.

### 3.3. Using machine learning to identify biomarkers associated with disease extent

#### 3.3.1. Using the OPLS-DA model to screen differential metabolites

Next, we analyzed the differences in the serum metabolic profiles of patients with UC with different disease extents. Figure S1 shows OPLS-DA score scatter plots and permutation test plots. There were 69 (33 upregulated and 36 downregulated), 82 (26 upregulated and 56 downregulated), and 57 (17 upregulated and 40 downregulated) differential metabolites obtained from the OPLS-DA model in the E1 and E2, E1 and E3, and E2 and E3 groups, respectively (Table S2). However, the OPLS-DA model had varying degrees of overfitting, which means that the model cannot effectively identify differential metabolites.

#### 3.3.2. Identification of key metabolites by machine learning

To identify the key metabolites related to the disease extent of UC and avoid overfitting, we used the LASSO, RF-RFE, and SVM-RFE algorithms to screen the 913 detected metabolites. Finally, there were 23, 6, and 6 differential metabolites obtained by machine learning in the E1 and E2, E1 and E3, and E2 and E3 groups, respectively.

**Figure 5.**
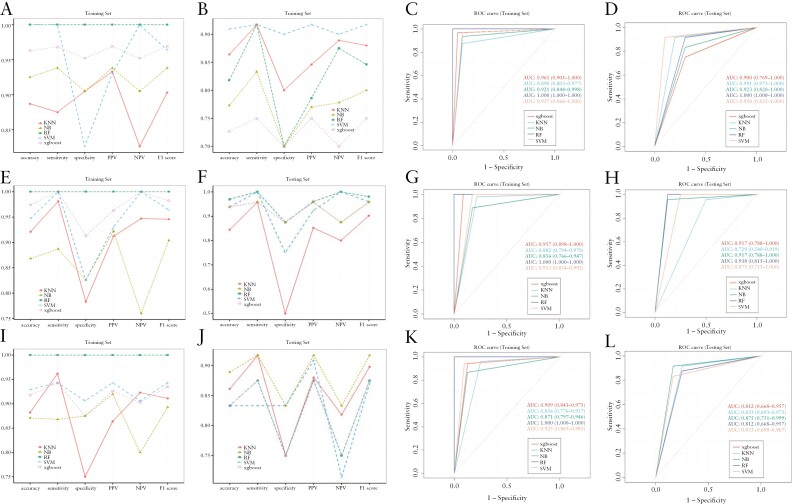
(A,E,I) The accuracy, sensitivity, specificity, PPV, NPV, and F1 score of the training set prediction model of group E1 vs E2,E1 vs E3, and E2 vs E3, respectively. (B,F,J) The accuracy, sensitivity, specificity, PPV, NPV, and F1 score of the testing set prediction model of group E1 vs E2,E1 vs E3, and E2 vs E3, respectively. (C,G,K) The ROC curve of the training set prediction model of group E1 vs E2, E1 vs E3, and E2 vs E3, respectively. (D,H,L) The ROC curve of the testing set prediction model of group E1 vs E2, E1 vs E3, and E2 vs E3, respectively. AUC, area under the receiver operating characteristic curve; KNN, K-NearestNeighbor; NB, Naive Bayes; NPV, negative predictive value; PPV, positive predictive value; RF, random forest; ROC, receiver operating characteristic; SVM, support vector machine; Xgboost, eXtreme Gradient Boosting.

Based on the above metabolites, we constructed prediction models for the extent of UC and found that all 5 models performed well. Among them, the prediction accuracy of the RF prediction model in the 3 training sets was as high as 100%, and the AUCs were all 1. Even in the testing set, the prediction probability of the RF model was high, and the AUCs were 0.808 (95% CI: 0.639-0.979), 0.938 (95% CI: 0.815-1.000), and 0.812 (95% CI: 0.668-0.957) (Figure 5). In the training set, the AUCs of all prediction models are greater than 0.85, and in the testing set, the AUCs are greater than 0.7. This result indicates that key metabolites can effectively predict the disease extent of UC.

**Figure 6.**
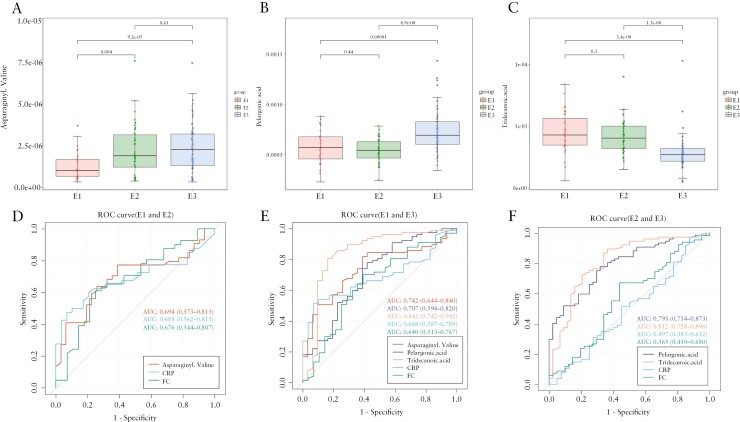
(A) The inter-group comparison box chart of asparaginyl valine. (B) The inter-group comparison box chart of pelargonic acid. (C) The inter-group comparison box chart of tridecanoic acid. (D) ROC curve for distinguishing biomarkers between E1 and E2 groups. (E) ROC curve for distinguishing biomarkers between E1 and E3 groups. (F) ROC curve for distinguishing biomarkers between E2 and E3 groups. CRP, C-reactive protein; FC, fecal calprotectin; ROC, receiver operating characteristic.

However, these metabolites can only reflect inter-group differences. Therefore, we compared 3 groups of differential metabolites and found that the serum level of pelargonic acid in patients with extensive colitis was significantly higher than that in the other 2 groups (*P* < .000), and the serum level of tridecanoic acid was significantly lower than that in the other 2 groups (*P* < .000). The serum level of asparaginyl valine in patients with rectal UC was significantly lower than that in patients in the E2 and E3 groups (Figure 6A-C). This result suggests that asparaginyl valine is associated with local inflammation in the rectum and can serve as a potential biomarker for predicting E1, while tridecanoic acid and pelargonic acid are associated with extensive colitis and can serve as potential biomarkers for predicting E3.

Then, to clarify the ability of the above 3 substances to distinguish different disease extents, we also established ROC curves. And compared it with established biomarkers such as C-reactive protein (CRP) and fecal calprotectin (FC). We found that the asparaginyl valine (AUC 0.694 [0.573-0.815]) did not show an advantage over CRP (AUC 0.688 [0.562-0.815]) and FC (AUC 0.676 [0.544-0.807]) when distinguishing between E1 and E2 groups. However, asparaginyl valine (AUC 0.742 [0.644-0.840]), pelargonic acid (AUC 0.707 [0.594-0.820]), and tridecanoic acid (AUC 0.842 [0.742-0.942]) performed significantly better than CRP (AUC 0.688 [0.587-0.789]) and FC (AUC 0.640 [0.513-0.767]) when distinguishing between E1 and E3 groups. When distinguishing between the E2 and E3 groups, pelargonic acid (AUC 0.793 [0.714-0.813]) and tridecanoic acid (AUC 0.812 [0.728-0.892]) also performed very well (Figure 6D-F). Therefore, we suggest that pelargonic acid and tridecanoic acid have a close relationship with proximal intestinal inflammation and can be used as biomarkers to predict widespread intestinal inflammation.

## 4. Discussion

UC is a metabolism-related inflammatory disease. It is widely believed that genetic, environmental, dietary, and psychological factors interact to cause intestinal microbial metabolism disorders and participate in the complex mechanism of IBD.^21^ Previous studies have also confirmed that patients with UC have obvious metabolic disorders compared with healthy people.^22^

Our study found that tryptophan metabolism, glycerophospholipid metabolism, and the PPAR signaling pathway were disordered in patients with UC, consistent with previous research. Tryptophan is an essential amino acid in intestinal mucosal cells that participates in intestinal immune inflammation and is closely related to the pathogenesis of IBD. Trp is metabolized by different pathways to a variety of indole derivatives, 5-hydroxytryptamine (5-HT), D-tryptophan, and other products. Its metabolic end products are crucial for intestinal homeostasis^23^ and are common therapeutic targets in cancer, neurodegeneration, and other diseases.^24^ The tryptophan metabolic pathway mainly includes 3 metabolic pathways, namely, the kynurenine (Kyn) pathway, 5-HT pathway, and indole pathway.^25^ The Kyn pathway is the main tryptophan metabolic pathway. Studies have confirmed that Kyna levels during remission in UC and CD are lower than those during recurrence.^26,27^ Chloé’s study showed that the severity of intestinal inflammation in UC patients is negatively correlated with the content of xanthinate and kynurine, and supplementation with xanthinate or kynurine can reduce intestinal inflammation.^28^

Glycerophospholipid metabolism is also thought to be closely related to UC. Kun Liu et al. applied metabolomics techniques to identify biomarkers for evaluating the efficacy of mesalazine as a treatment for UC and found that IBD dysregulation was related to primary bile acid biosynthesis, arachidonic acid metabolism, sphingolipid metabolism, fatty acid elongation, and glycerophospholipid metabolism.^29^ An experimental animal study also found that the traditional Chinese medicine Huanglian Jiedu Decoction can alleviate UC in mice by regulating glycerophospholipid metabolism.^30^

PPAR is a member of the superfamily of ligand-activated nuclear transcription factors and includes 3 phenotypes, PPAR-α, PPAR-β/δ, and PPAR-γ, of which PPAR-γ is the most thoroughly studied.^31^ It was found that PPAR-γ synthesis is low in the epithelial cells of mice with UC and dextran sodium sulfate-induced colitis.^32,33^ Analysis of the colon of patients with UC by reverse transcription-polymerase chain reaction (RT-PCR), western blotting, and immunohistochemical methods showed a decrease in PPAR-γ mRNA and protein levels compared with those in HCs.^34^ Yamamoto-Furusho et al. also reported a decrease in the mRNA expression of PPAR-γ in the mucosa of patients with active UC compared with that in patients with UC who were in remission, suggesting that PPAR-γ expression is inversely associated with UC progression.^35^ The Gegen Qinlian Decoction can improve colitis in mice by activating the PPAR-γ signal, which inhibits the expansion of *Enterobacteriaceae*.^36^ In recent years, a variety of drugs have been shown to improve intestinal inflammation by regulating the PPAR signaling pathway, and PPARs have also become experimental targets for the treatment of IBD.^31^

Clinically, disease activity greatly affects treatment decisions, and high levels of disease activity are closely associated with adverse outcomes in UC. Therefore, numerous clinical studies of UC have focused on disease activity, neglecting disease extent. Recent mechanistic studies of IBD, particularly genotypic studies of patients with CD at different sites, have made us realize that disease extent is also an intrinsic aspect of UC with important clinical significance.

In this study, we explored the metabolic profiles of patients with UC and identified potential biological correlates of UC in patients with different disease extents by combining metabolomics and machine learning. Owing to the severe overfitting of the OPLS-DA model when applied to inter-group discrimination, we adopted a machine learning approach to screen metabolites and construct predictive models. We found that certain metabolites have great potential in predicting the location of disease lesions, and the predictive performance of the models remained satisfactory even when the sample size was small. We also analyzed 3 groups of differential metabolites and found that the serum level of pelargonic acid in patients with extensive colitis was significantly higher than that in the other 2 groups, and the serum level of tridecanoic acid was significantly lower. Both pelargonic acid and tridecanoic acid are saturated fatty acids with medium-length chains, and neither has been studied in depth. Research suggests that nonanoic acid upregulates endogenous host defense peptides and enhances intestinal epithelial immune barrier function by inhibiting histone deacetylase.^37^ Some studies have found that tridecanoic acid can inhibit *Escherichia coli* persistent cell formation and repress biofilm activity.^38^ Another study found that the content of tridecanoic acid in patients with lung cancer was significantly lower than that in the HC group. The accuracy of identifying lung cancer using tridecanoic acid combined with 4 other metabolites can reach 82.9%.^39^ These metabolites have great potential as indicators of intestinal inflammation proximal progression in patients with UC. Our previous retrospective study also found that the levels of low-density lipoprotein cholesterol, high-density lipoprotein cholesterol, apolipoprotein A1, apolipoprotein E, and total cholesterol in patients with distal colitis were significantly higher than those in patients with extensive colitis.^13^ However, no other studies have linked the above pathways to the disease extent of UC. The relationship between these metabolic pathways and the disease extent of UC requires further research to refine the study of UC site phenotypes, facilitate drug development, and guide clinical drug use.

This study confirmed that the serum metabolomics of patients with different UC disease extents differed at the same level of disease activity. Although we lack an explanation for this observation, we propose 2 ways of considering it. First, the variation in serum metabolomics may be caused by variations in the different ranges of intestinal inflammation. This suggests that the screened metabolites can be helpful for predicting both the range of intestinal lesions in UC and disease progression. Second, the variation in serum metabolomics may be related to the intrinsic molecular structure of the colon and the molecular mechanism of intestinal inflammation in UC. In clinical practice, many drugs for treating UC are selective targeted therapies, and biological agents are representative drugs. Further exploration into the molecular differences of patients with UC may facilitate the development of clinical precision medicine.

This study has some limitations. Our study is the first to examine the differences in metabolites in patients with different disease extents, and the results lack evidentiary support. Additionally, this study used only serum samples for metabolomics detection; subsequent multi-sample and multi-omics studies are required for cross-validation. Therefore, we plan to collect intestinal mucosa samples from patients with UC at different sites to further validate our findings.

## Supplementary Material

jjaf020_suppl_Supplementary_Material

## Data Availability

The data underlying this article are available in the article and in its online supplementary material. The data supporting the conclusions of this article are included within the article. The manuscript, including related data, figures, and tables, has not been previously published, and it is not under consideration elsewhere.
