# Supplementary material for: Machine learning and metabolomics identify biomarkers associated with the disease extent of ulcerative colitis
Source: J Crohns Colitis. 2025 Feb 4;19(2):jjaf020. doi: 10.1093/ecco-jcc/jjaf020 (PMC11829215; doi:10.1093/ecco-jcc/jjaf020)
Supplement: jjaf020_suppl_Supplementary_Material [file jjaf020_suppl_supplementary_material.zip › Supplementary figure.pptx]

## Slide 1
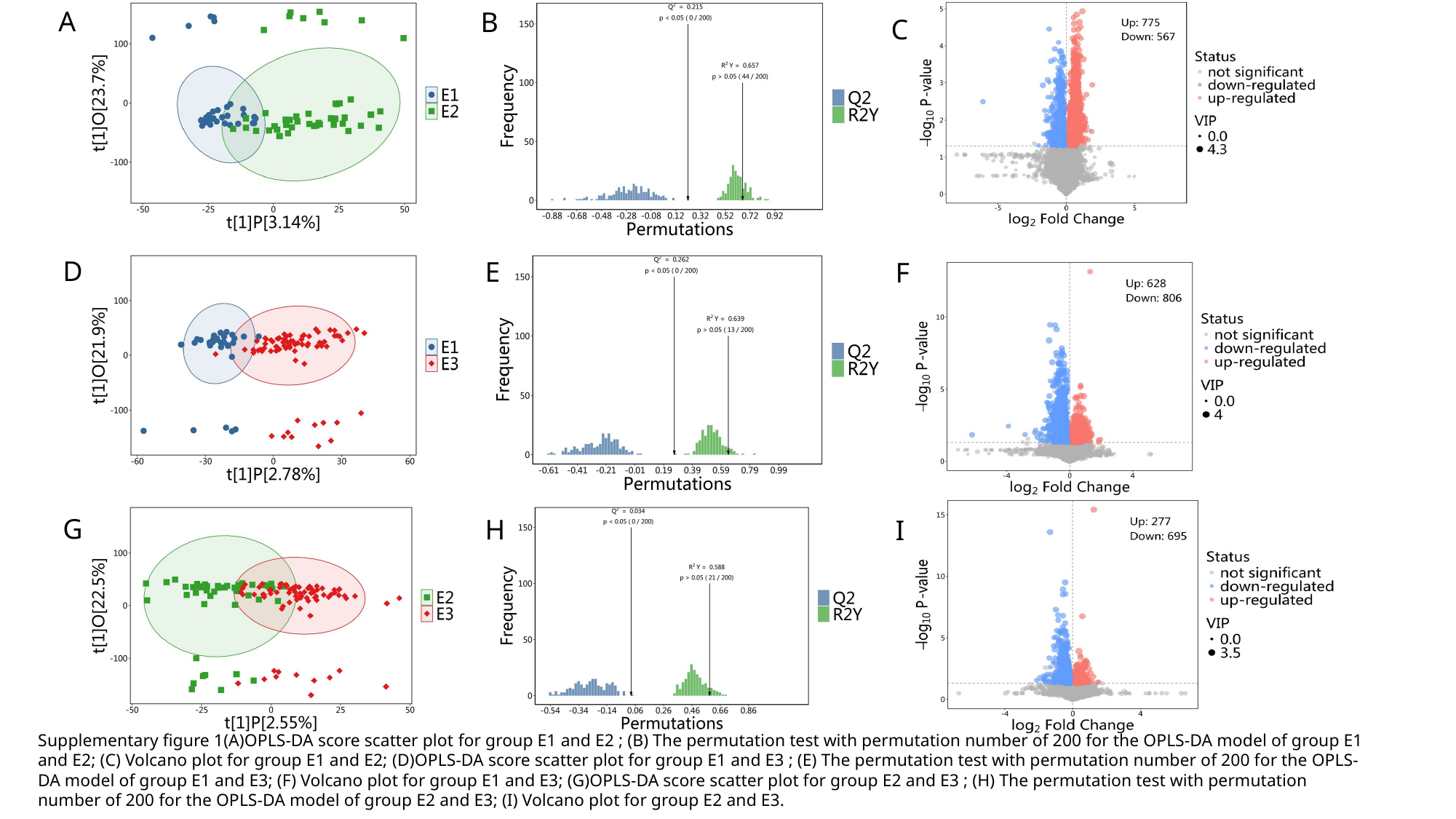

A
B
C
D
E
F
G
H
I
Supplementary figure 1(A)OPLS-DA score scatter plot for group E1 and E2 ; (B) The permutation test with permutation number of 200 for the OPLS-DA model of group E1 and E2; (C) Volcano plot for group E1 and E2; (D)OPLS-DA score scatter plot for group E1 and E3 ; (E) The permutation test with permutation number of 200 for the OPLS-DA model of group E1 and E3; (F) Volcano plot for group E1 and E3; (G)OPLS-DA score scatter plot for group E2 and E3 ; (H) The permutation test with permutation number of 200 for the OPLS-DA model of group E2 and E3; (I) Volcano plot for group E2 and E3.
